# Aerobic Exercise Training Adaptations Are Increased by Postexercise Carbohydrate-Protein Supplementation

**DOI:** 10.1155/2011/623182

**Published:** 2011-06-09

**Authors:** Lisa Ferguson-Stegall, Erin McCleave, Zhenping Ding, Phillip G. Doerner III, Yang Liu, Bei Wang, Marin Healy, Maximilian Kleinert, Benjamin Dessard, David G. Lassiter, Lynne Kammer, John L. Ivy

**Affiliations:** Exercise Physiology and Metabolism Laboratory, Department of Kinesiology and Health Education, University of Texas at Austin, Austin, TX 78712, USA

## Abstract

Carbohydrate-protein supplementation has been found to increase the rate of training adaptation when provided postresistance exercise. The present study compared the effects of a carbohydrate and protein supplement in the form of chocolate milk (CM), isocaloric carbohydrate (CHO), and placebo on training adaptations occurring over 4.5 weeks of aerobic exercise training. Thirty-two untrained subjects cycled 60 min/d, 5 d/wk for 4.5 wks at 75–80% of maximal oxygen consumption (VO_2_ max). Supplements were ingested immediately and 1 h after each exercise session. VO_2_ max and body composition were assessed before the start and end of training. VO_2_ max improvements were significantly greater in CM than CHO and placebo. Greater improvements in body composition, represented by a calculated lean and fat mass differential for whole body and trunk, were found in the CM group compared to CHO. We conclude supplementing with CM postexercise improves aerobic power and body composition more effectively than CHO alone.

## 1. Introduction

It is well established that aerobic exercise training leads to cardiovascular, skeletal muscle, and metabolic adaptations. Cardiovascular adaptations include increased stroke volume and cardiac output, which contributes greatly to increased maximal oxygen consumption (VO_2_ max) [1, 2]. Skeletal muscle adaptations include increases in activators of mitochondrial biogenesis such as peroxisome proliferator-activated receptor *γ* coactivator-1*α* (PGC-1*α*), and increased activity of oxidative enzymes such as citrate synthase and succinate dehydrogenase [3–10]. While many investigations have addressed the effects of endurance exercise training on such adaptations, few have examined the role of postexercise nutritional supplementation in facilitating the adaptive process. 

The beneficial effects of postexercise supplementation in the form of carbohydrate (CHO) or carbohydrate-protein (CHO+PRO) supplements following an acute exercise bout have been the focus of many investigations. Several studies performed by our laboratory, and others have demonstrated a greater improvement in acute exercise recovery with CHO+PRO supplementation compared to CHO alone [11–14] or to placebo [15]. Okazaki and colleagues [16] recently compared the effects of a CHO+PRO supplement to a placebo supplement in older male subjects who cycled for 60 min/d, 3 d/wk for 8 wk at 60–75% VO_2_ peak. They reported a twofold increase in VO_2_ max in the CHO+PRO group compared to the placebo group [16]. Thus, nutritional supplementation may increase the magnitude of training adaptations compared to the exercise stimulus alone. However, it was not possible to determine from their results if the increase in VO_2_ max was due to cellular or systemic adaptations. Moreover, their experimental design did not allow for macronutrient specific comparisons, as they did not include a CHO-only supplement. 

Recently, chocolate milk (CM) has been investigated as a practical and effective CHO+PRO postexercise recovery supplement after aerobic exercise [17–19]. In addition, several investigations have reported the efficacy of milk-based supplements in increasing protein synthesis [20] and lean mass accrual [19, 21, 22] in response to resistance exercise. However, the effects of aerobic endurance exercise training and nutritional supplementation on body composition changes have not been investigated.

Therefore, the purpose of the present study was to investigate training adaptations that occurred after a 4.5 wk aerobic endurance exercise (cycling) training program when supplementing after each daily exercise session with a CHO+PRO supplement in the form of CM, CHO or placebo. We aimed to determine if nutritional supplementation resulted in a greater increase in VO_2_ max and skeletal muscle oxidative enzyme activity. We also sought to determine if supplementation resulted in a greater increase in lean mass and a greater decrease in fat mass. Although the exercise training was expected to induce positive adaptations in VO_2_ max and muscle oxidative capacity, we hypothesized that postexercise CM supplementation would induce a greater extent of adaptations than would occur with CHO or PLA supplementation. We further hypothesized that the CM group would demonstrate greater lean body mass increases and fat mass decreases compared to the CHO and PLA groups.

## 2. Materials and Methods Lactate Threshold and VO_2_ max

Diet and Exercise

Subjects were asked to keep their diets and activity levels consistent for the duration of the study (i.e., no significant changes in caloric intake, dietary habits, or activity levels outside of the study's training sessions). The subjects were instructed to maintain a dietary and activity log for the 2 days prior to their baseline and end biopsies and testing. The subjects were also asked to replicate their diet and activity on the days the logs are kept such that the diet and activity was the same on the 2 days prior to each biopsy session. The self-reported activity was compared for consistency in duration and intensity. The diet logs were analyzed for macronutrient composition and total caloric intake using Nutritionist V Dietary Analysis Software (First Data Bank, Inc, San Bruno, Calif, USA). All subjects complied with the diet and activity requirements. Subjects were instructed to arrive at the laboratory having fasted overnight for 12 h for every exercise session and laboratory visit except for the LT and VO_2_ max testing sessions.

These measures were determined at baseline and at the end of the 4.5 wk training period, as shown in Table 2. The protocol for these tests is detailed above.

### 2.1. Subjects

Thirty-two healthy, recreationally active but untrained males and females (16 males and 16 females) between 18 and 35 years old completed the study. Subject characteristics are listed in Table 1. In order to be classified as recreationally active but not endurance trained, subjects could not have exercised regularly more than 3 h/wk over the last 2 years, and had VO_2_ max values of <40 mL/kg/min for females and <45 mL/kg/min for males. Potential subjects who did not meet these criteria were screened out of the study. A total of 36 subjects were admitted to the study. Subjects were separated into three groups matched for age, gender, and body composition and then were randomized into one of the 3 treatment groups. Four subjects voluntarily withdrew due to illness or work scheduling conflicts. Written informed consent was obtained from all subjects, and the study was approved by The University of Texas at Austin Institutional Review Board.

### 2.2. Research Design

This study followed a randomized, double-blinded, placebo-controlled design. The protocol for the training period is shown in Table 2. The entire protocol period was 7 wk long and consisted of the following: a baseline testing week, the first and second weeks of training, a midpoint testing week in which subject's VO_2_ max was reassessed, followed by 2 days of training, training weeks 3 and 4, and a partial week during which the end testing was performed. Subjects reported to the laboratory before the start of their training period on two occasions, once for a baseline biopsy and dual energy X-ray absorbency (DEXA) scan for body composition determination (described below), and again the following day for determination of lactate threshold (LT), maximal oxygen consumption (VO_2_ max) and maximal workload (*W*_max_). This same test battery and schedule was repeated at the end of the training period (Table 2). 

The LT test was performed first, followed by the VO_2_ max test after a 5-min cool-down between the two tests. These tests were performed on a VeloTron DynaFit Pro cycle ergometer (RacerMate, Seattle, Wash, USA). LT was determined using 5-min stages beginning at 70 Watts (W) for males and 50 W for females. The Watts were increased by 25 W (males) or 20 W (females) each stage for the first 3-4 stages, followed by increases of 15 W (males) or 10 W (females) for the last 2-3 stages. A drop of blood was collected onto a lactate test strip after a finger stick during the last minute of each stage, and lactate levels were measured using a Lactate Pro LT-1710 lactate analyzer (Arkray, Inc., Minami-ku, Kyoto, Japan). LT was defined as the breakpoint at which lactate levels begin to rise above baseline levels. After the 5-min cool-down in which the subjects pedaled easily and drank water *ad libitum*, the VO_2_ max test began. VO_2_ max was measured using a True One 2400 system (ParvoMedics, Sandy, UT). Subjects breathed through a Hans Rudolph valve, with expired gases directed to a mixing chamber for analysis of oxygen (O_2_) and carbon dioxide (CO_2_). Outputs were directed to a computer for calculation of ventilation, O_2_ consumption (VO_2_), CO_2_ production (VCO_2_), and respiratory exchange ratio (RER) every 15 s.

The protocol for establishing VO_2_ max consisted of 2 min stages beginning at 125 W for males or 75 W for females. The workload was increased by 50 W (males) or 30 W (females) every 2 min until 275 W and 200 W, respectively. After this point, the workload increased 25 W (males) or 20 W (females) every minute until the subject could not continue to pedal despite constant verbal encouragement. The criteria used to establish VO_2_ max was a plateau in VO_2_ with increasing exercise intensity and RER > 1.10. 

Maximum power output in Watts was calculated from the VO_2_ max test data using the formula adapted from Åstrand and Rodahl [23]:

(1) $$W_{\max\;}\;=\left({\text{VO}}_{2\;}\max\;\;\text{mL}-300\;\text{mL}\;{\text{O}}_{2}\right)/12.5\text{W}/\text{mL}\;{\text{O}}_{2}.$$

The workload for the desired intensity level of the training rides (75% of VO_2_ max for the first 3.5 weeks and 80% for week 4) was then set as percentages of the *W*_max_ as follows:

(2) $$\begin{array}{cc} W & =\;[\left({\text{VO}}_{2\;}\max\;\;\text{mL}\times \%{\text{VO}}_{2}\;\max\;\;\text{desired}\right)\\ & \;\;-300\;\text{mL}\;{\text{O}}_{2}]\;/\;12.5\;\text{W}/\text{mL}\;{\text{O}}_{2}. \end{array}$$

With the exception of determining *W*_max_ for the purposes of setting ride intensity levels, the baseline and end testing consisted of the same tests in the same order. 

During the training weeks, subjects reported to the training laboratory each morning after fasting overnight. All subjects began the rides as a group at the same time each day (6:00 AM or 7:30 AM), Monday–Friday. After each session, subjects were provided one dose of supplement immediately postexercise and were required to drink it in the laboratory. Subjects were then provided a second dose in an opaque to-go cup with a lid and straw and instructed to drink it 1 h later. They were also instructed to not ingest anything other than water until 1 h after ingesting the second dose. 

The daily training rides were performed on Kona Dew bicycles (Kona, Ferndale, Wash, USA) mounted on CompuTrainer stationary trainers (RacerMate, Seattle, Wash, USA) interfaced with MultiRider III software (RacerMate, Seattle, Wash, USA). Six bicycles and CompuTrainers were interfaced with the system to allow for training groups of 6 subjects at one time. The bikes were set up based on each subject's physical measurements. The CompuTrainers were calibrated each morning. To minimize thermal stress, air was circulated over the subjects with standing floor fans, and water was provided ad libitum. Investigators encouraged the subjects to drink as needed.

The first week of training served to get the subjects accustomed to cycling for prolonged periods. The first ride was 30 min in duration, the second was 40 min, the third ride, 50 min, and the fourth, 60 min. With the exception of 3 rides the first week, all rides on Monday–Friday were 1 h in duration throughout the training period. 

Each training ride began with a 10-min warm up at 60% VO_2_ max, after which the work rate was increased to elicit ~75% VO_2_ max for duration of each training ride. At the midpoint, VO_2_ max was reassessed, and the workloads were adjusted accordingly to keep the subjects exercising at 75% VO_2_ max for the third week. For the fourth week, the intensity was increased to 80% VO_2_ max. A 5-min VO_2_ measurement was performed at the beginning of each week to verify that the workload corresponded to the calculated intensity (%VO_2_ max) for each subject. The Wattage calculated for each subject was set by the investigators, and subjects were asked to maintain a cadence of ~70 rpms in order to maintain the Wattage. Subjects were not allowed to shift gears or vary their cadence during the rides. The duration of the training period (4.5 weeks) was chosen, because 4 weeks or less has been shown to be an adequate amount of time to demonstrate VO_2_ max and oxidative enzyme activity changes [13, 16].

### 2.3. Experimental Beverages

After each daily session, subjects ingested the experimental beverages (CM, CHO, or PLA) immediately and l h postexercise. The CM (Kirkland Organic Low-Fat Chocolate Milk, Costco Inc.) and CHO beverages were isocaloric and contained the same amount of fat. The placebo was an artificially flavored and artificially sweetened supplement that resembled the CHO beverage in taste and appearance but contained no calories. Grape-flavored Kool-Aid was selected for the CHO and PLA treatments, because it best matched the dark coloring of the CM treatment visible only through a semiopaque lid on the drink containers. The energy and macronutrient composition of the beverages is shown in Table 3.

The amounts of supplement provided were stratified according to body weight ranges. Subjects weighing less than 63.6 kg (140 lbs) received 250 mL per supplement (197.5 kcals each), totaling 500 mL and 395 kcals. Subjects weighing between 63.6 kg (140 lbs) and 77.2 kg (170 lbs) received 300 mL per supplement (237 kcals), totaling 600 mL and 474 kcals. Subjects weighing between 77.2 kg (170 lbs) and 90.9 kg (200 lbs) received 350 mL per supplement (277 kcals), totaling 700 mL and 554 kcals. Subjects weighing over 90.9 kg (200 lbs) received 375 mL per supplement (296.5 kcals), totaling 750 mL and 593 kcals. For the CHO treatment, the amount of carbohydrate (dextrose) and fat (canola oil) matched that provided in the CM as measured for the individual's weight range. The CM supplement provided an average of 0.94 g carbohydrate, 0.31 g protein, and 0.17 g fat per kg body weight. The CHO supplement provided an average of 1.25 g carbohydrate and 0.17 g fat per kg body weight.

### 2.4. Muscle Biopsy Procedure

Muscle biopsies were taken at baseline and the end of the training period, as shown in Table 2. Prior to each biopsy, the subject's thigh was cleansed with 10% betadine solution and 1.4 mL of 1% Lidocaine Hydrochloride (Elkins-Sinn, Inc., Cherry Hill, NJ) was injected to prepare the leg for the muscle biopsy. Approximately ~45–60 mg wet wt of tissue was taken from the vastus lateralis through a 5–8 mm incision made through the skin and fascia, 6 inches from the midline of the thigh on the lateral side and 2.5 inches above the patella. The tissue samples were trimmed of adipose and connective tissue and immediately frozen in liquid nitrogen at −80°C for subsequent analysis.

### 2.5. Muscle Tissue Processing

The muscle samples were weighed and cut in half. One half of the tissue sample was used for the determination of citrate synthase and succinate dehydrogenase activity, and the other half for measurement of total PGC-1*α* content. For the enzymatic analyses, samples were homogenized in ice-cold buffer containing 20 mM Hepes, 2 mM EGTA, 50 mM sodium fluoride, 100 mM potassium chloride, 0.2 mM EDTA, 50 mM glycerophosphate, 1 mM DTT, 0.1 mM PMST, 1 mM benzamidine, and 0.5 mM sodium vanadate (pH 7.4) at a dilution of 1 : 10. Homogenization was performed on ice using 3×5 s bursts with a Caframo RZRl Stirrer (Caframo Limited, Warton, Ontario, Canada). The homogenate was immediately centrifuged at 14,000 g for 10 min at 4°C, the supernatant aliquoted to storage tubes for each assay and stored at −80°C. For determination of total PGC-1*α* content, the tissue samples were homogenized at a dilution of 1 : 10 in a modified RIPA buffer based on a previously described protocol [24] containing: 50 mM Tris-HCL (pH 7.4); 150 mM NaCl (pH 7.4); 1% each Igepal CA-630 and sodium deoxycholate; 1 mM each EDTA (pH 7.4), Na_3_VO_4_ (pH 10), NaF, and phenylmethylsulfonyl fluoride; 1 *μ*g/mL each aprotinin, leupeptin, and pepstatin. Homogenization was performed on ice using 4×5 s bursts with a Caframo RZRl Stirrer (Caframo Limited, Warton, Ontario, Canada). The homogenates were sonicated on ice for 10 s and then centrifuged at 5,000 g for 20 min at 4°C. The supernatant was aliquoted to storage tubes and stored at −80°C. Protein concentration was determined from the supernatant using a modified version of the Lowry assay [25] for each sample and was measured before each of the assays were performed.

**PGC-1*α* Content**

Total PGC-1*α* content was determined by Western blotting. Total *α*-tubulin content was also determined as a housekeeping protein. Aliquots of homogenized muscle sample supernatants and standards were slowly thawed over ice and diluted 1 : 1 with sample buffer containing 1.25 M Tris, pH 6.8, glycerol, 20% SDS, 2-mercaptoethanol, 0.25% bromophenol blue solution, and deionized water. Samples containing 70 *μ*g of total protein were separated on 10% polyacrylamide gels by SDS-PAGE for 75 min at 200 V (Bio-Rad Laboratories, Hercules, Calif, USA) After electrophoresis, the gels were electrotransferred using a semi-dry transfer cell (Bio-Rad Laboratories, Hercules, Calif, USA) using 25 V for 18 min to 0.4 *μ*m polyvinylidene fluoride (PVDF) membranes (Millipore, Bedford, Mass, USA). The membranes were blocked in TTBS (TBS, 50 mM Tris, 150 mM NaCl, containing 0.1% Tween-20), and 10% nonfat dry milk for 2 h at room temperature on a rocking platform at medium speed. The membranes were then washed in 1x TTBS 3 times for 5 min each wash. Using the molecular weight markers visible on the membranes as a guide, the membranes were cut at the 75 kD marker. The upper section was used for detection of PGC-1*α*, and the lower section was used to detect *α*-tubulin. Each membrane section was incubated overnight at 4°C on a rocking platform at low speed with antibodies directed against PGC-1*α* (no. 515667, EMD Calbiotech/Merck KGaA, Darmstadt, Germany), and *α*-tubulin (no. 2144, Cell Signaling, Danvers, Mass, USA). The antibodies were diluted 1 : 1000 for PGC-1*α*, and 1 : 900 for *α*-tubulin in TTBS containing 2% nonfat dry milk. Following the overnight incubation, membranes were washed three times with TTBS for 5 min each wash and incubated for 1.5 h with a secondary antibody (goat antirabbit, HRP-linked IgG, no. 7074, Cell Signaling, Danvers, Mass, USA). Dilutions were 1 : 7500 for PGC-1*α* and 1 : 1000 for *α*-tubulin. The immunoblots were visualized by enhanced chemiluminescence (Perkin Elmer, Boston, Mass, USA) using a Bio-Rad ChemiDoc detection system, and the mean density of each band was quantified using Quantity One 1-D Analysis software (Bio-Rad Laboratories, Hercules, Calif, USA). A molecular weight ladder (Precision Plus Protein Standard, Bio-Rad) and a rodent internal control standard prepared from insulin-stimulated mixed skeletal muscle were also included on each gel. All blots were compared with the rodent control standard and the values of each sample were represented as a percent of standard for each blot.

### 2.6. Oxidative Enzymes

Citrate synthase (CS) activity was determined according to the protocol of Srere [26] on the homogenates after further dilution of 1 : 10 (wt/vol) with 0.1 M Tris-HCI and 0.4% Triton X-100 buffer (pH 8.1). The rate of appearance of DTNBwas determined spectrophotometrically over 5 min at 412 nm and 37°C using a Beckman DU 640 spectrophotometer (Fullerton, Calif, USA), and was proportional to CS activity. Succinate dehydrogenase (SDH) activity was measured according to the method of Lowry and Passonneau [27]. The amount of NADH produced during a 5 min incubation time was read on a Varian Cary Eclipse fluorometer with an excitation wavelength of 340 nm and emission wavelength of 450 nm (Varian, Inc., Palo Alto, Calif, USA) and corresponded to SDH activity in the sample. CS and SDH activities were expressed as *μ*mol/g/min protein.

### 2.7. Body Composition

DEXA (Medical Systems Prodigy, General Electric, Madison, Wis, USA) was used to determine both whole body and regional (trunk and legs) changes in fat mass, and lean mass, as well as bone mineral density (BMD). A three-compartment model design for assessing body composition was used, dividing the body into bone, fat mass, and fat-free mass. The total region percentage of fat mass and lean mass were used to assess the subjects' body fat and lean mass levels. The trunk region and legs region were used to assess fat and lean mass changes in the trunk and legs independently. The DEXA machine was calibrated each morning prior to subject measurement. Measurements were performed at baseline and at the end of the training period. The same trained technician performed all of the DEXA scans for the entire study.

The body composition differentials (Figures 4(a), 4(b), and 4(c)) were calculated according to the formula

(3) $$\begin{array}{cc} & \left({\text{LMkg}}_{\text{End}}-{\text{LMkg}}_{\text{Baseline}}\right)-\left({\text{FMkg}}_{\text{End}}-{\text{FMkg}}_{\text{Baseline}}\right)\\ & \;\;=\text{Differential}\;\left(\text{kg}\right). \end{array}$$

Using this formula, a gain in lean mass, and a loss of fat mass would result in a higher differential value than a loss in lean mass and gain in fat mass, or no change in lean and fat mass. This differential was calculated for whole body as well as regional (trunk and legs) changes (Figures 4(a), 4(b), and 4(c)). Therefore, the whole body differential was calculated as follows, using the CM treatment group values as an example:

(4) 1.408 kg−(−1.363 kg)=2.771 kg.

The regional differentials were calculated by the same formula using the values from those specific regions.

### 2.8. Statistical Analyses

Using data in the literature similar to the type of study we proposed, a power analysis was performed using G-Power 3.0.10 software (Buchner, Erdfelder and Faul, Dusseldorf University, Germany) for an effect size of 0.3, *P* < .05, and desired power value of 0.8, using 3 treatment groups. A total sample population of 24 subjects was calculated for an actual power of 0.86 although we collected data on 32 subjects total.

VO_2_ max, LT, muscle enzyme activity, PGC-1*α* content and body composition measures (lean mass, fat mass, and weight) taken at baseline and end were analyzed using two-way (treatment x time) analysis of variance (ANOVA) for repeated measures. Differences in the baseline and end measurements for VO_2_ max, as well as forthe body composition differentials were analyzed using a one-way ANOVA. For all measures, post hoc analysis was performed when significance was found using least significant difference (LSD). Differences were considered significant at *P* < .05. Effect sizes were calculated for VO_2_ max changes and body composition differentials using the value of Cohen's *d* and the effect-size correlation. Data were expressed as mean ± SE. All statistical analyses were performed using SPSS version 16.0 statistical software (SPSS Inc., Chicago, Ill, USA).

## 3. Results

### 3.1. VO_2_ max and Lactate Threshold

**Figure 1:**
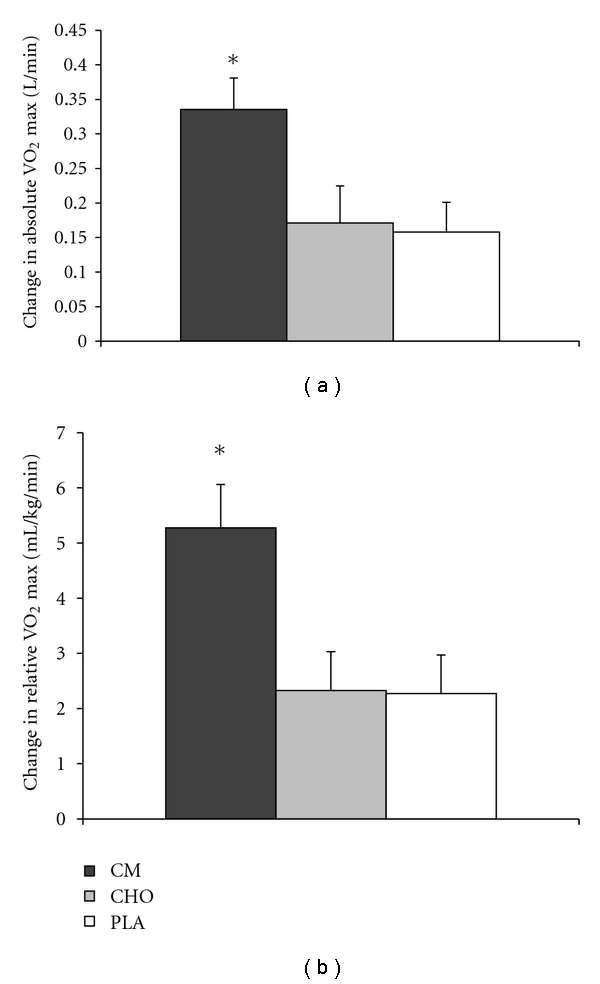
VO_2_ max changes after 4.5 wks of aerobic endurance training. (a) Change from baseline in absolute VO_2_ max. (b) Change from baseline in relative VO_2_ max. Values are mean ± SE. Significant treatment differences: *, CM versus PLA and CHO (*P* < .05).

Absolute and relative changes in VO_2_ max are shown in Figure 1. No significant differences existed between the groups at baseline. All treatment groups experienced significant increases in absolute and relative VO_2_ max over the 4.5 wk training period. The change in both absolute and relative VO_2_ max was significantly greater in the CM group compared to CHO (*P* < .05; absolute effect size, 0.86; relative effect size, 0.89) and PLA (*P* < .05; absolute effect size, 0.89; relative effect size, 0.90). The increases in the CHO and PLA groups were not statistically different from each other (Figure 1). LT increased significantly over time in all 3 treatment groups. However, there were no significant differences among the treatments (Table 4).

### 3.2. Oxidative Enzymes and PGC-1*α*

**Figure 2:**
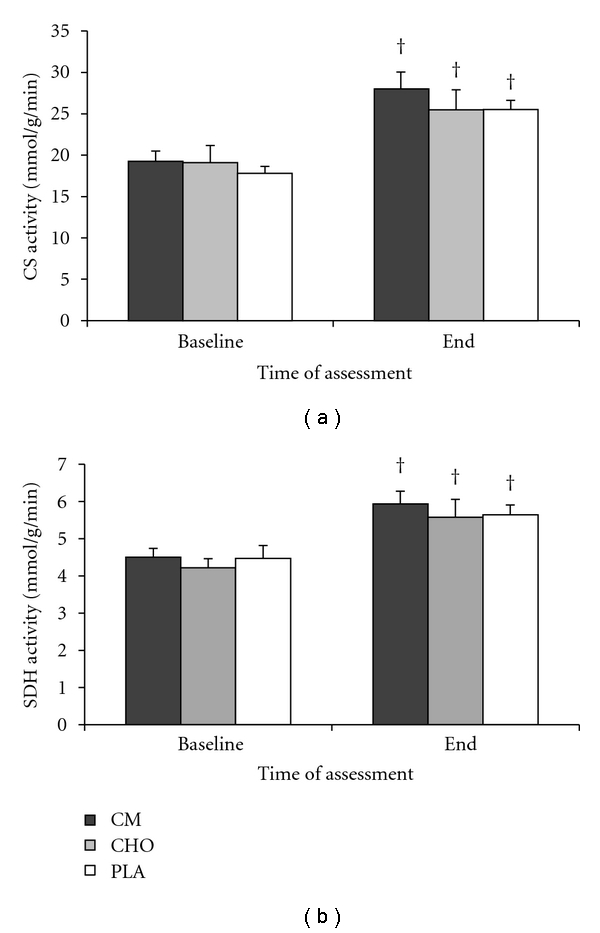
Oxidative enzyme activity. (a) Citrate synthase activity. (b) Succinate dehydrogenase activity. Biopsies were taken at baseline (before starting the training period) and at the end of the 4.5 wk training period. No significant treatment differences were found. †, significant time effect (*P* < .05).

**Figure 3:**
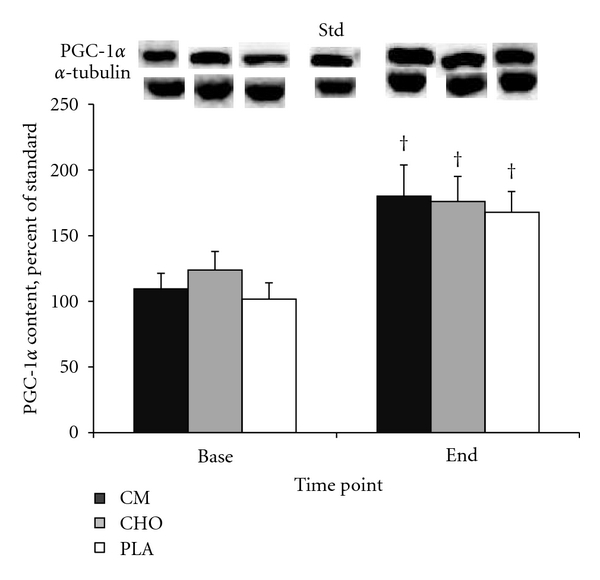
PGC-1*α* content before and after 4.5 wks of cycling exercise training. No significant treatment differences were found. ^†^, significant time effect (*P* < .05).

No significant treatment or treatment by time effects were found for CS, SDH (Figures 2(a) and 2(b)) or PGC-1*α* (Figure 3). Significant time effects existed for both enzymes in all treatment groups (*P* < .05). A similar response was found for PGC-1*α* (*P* < .05).

### 3.3. Body Composition

Changes in body weight, lean mass and fat mass (assessed for whole body, trunk, and legs) are shown in Table 5. Whole-body lean mass increased in all treatment groups, with no treatment differences detected (*P* < .05). Although whole-body fat mass decreased in all groups, the change was not significant for treatment or time. In the trunk region, a significantly greater gain in lean mass was found in the CM group compared to PLA (*P* < .05). Trunk region fat mass differences were not significantly different between treatments although a significant time effect was found for all groups (*P* < .05). In the legs region, significant time effects were found for lean mass increases and fat mass decreases in all groups (*P* < .05). 

**Figure 4:**
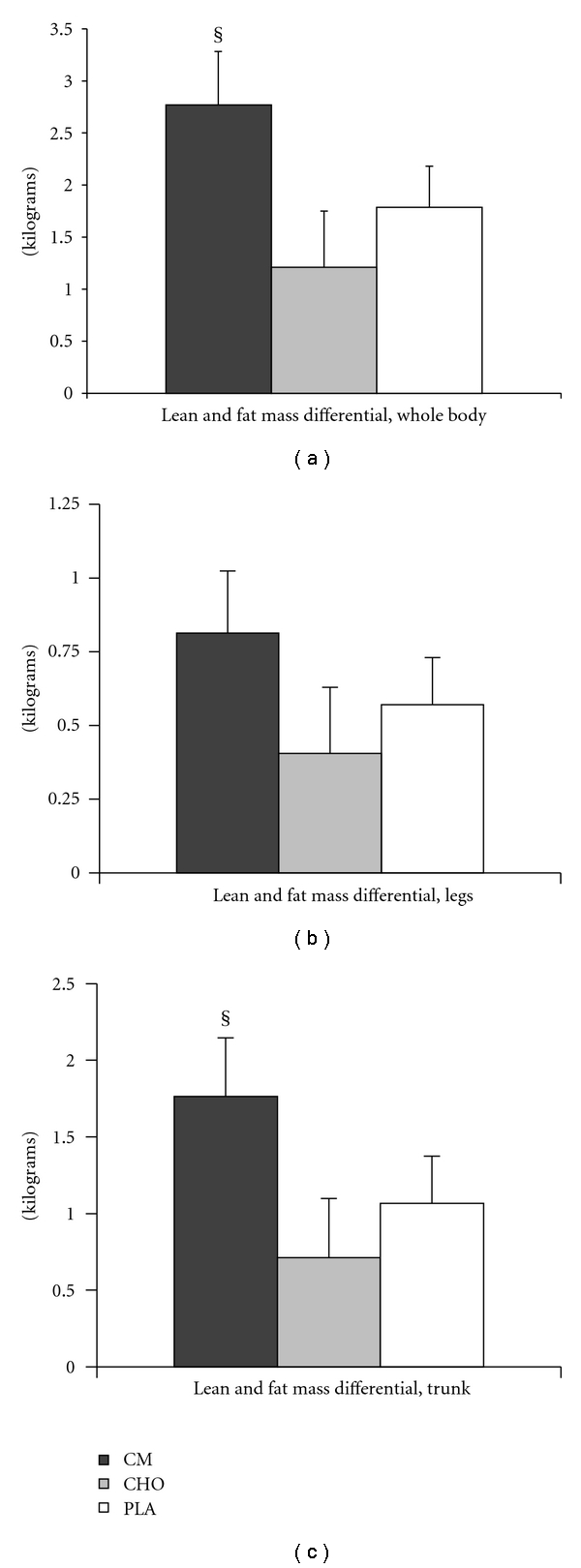
Body composition lean and fat mass differentials. (a) Whole body differential. Lean mass (kg) gained and fat mass (kg) lost was used to calculate a whole-body differential to quantify overall body composition changes in response to 4.5 wks of cycling exercise training. (LM) − (FM) = differential. Example: (0.900 kg lean mass) − (−0.350 kg fat mass) = 1.250 kg. (b) Lean and fat mass differential for the legs. (c) Lean and fat mass differential for the trunk region. Values are mean ± SE. Significant treatment differences: ^§^, CM versus CHO (*P* < .05).

The whole body and regional differentials are shown in Figures 4(a), 4(b), and 4(c). The whole body differential and the trunk differential were significantly greater in the CM group compared to CHO (*P* < .05; effect size for trunk, 0.81; effect size for whole body, 0.82). Whole body and trunk differentials for PLA were not significantly different than those for CM or CHO. The differential for the legs region was not significantly different among the three treatments. No significant treatment or time differences existed for BMD (Table 5).

## 4. Discussion

The most significant finding of the present study was that the increase in VO_2_ max was significantly greater in the CM group than the CHO or PLA groups. The average increase in absolute VO_2_ max for the CM group was 12.5% higher than baseline levels, a twofold improvement over the increase found in the CHO and PLA groups. The average absolute VO_2_ max (L/min) increase for all subjects and treatment groups combined was 9.2% over the 4.5 wk training period, which is in agreement with other investigations of aerobic training and VO_2_ max improvements using a similar time period [28, 29].

It has been established that the primary determinants of VO_2_ max are an increased ability of the cardiovascular system to transport oxygen to the working skeletal muscle, and the improved ability of the muscle to utilize the delivered oxygen. The former is a result of increased stroke volume, which improves cardiac output; the latter is determined by the increases in oxidative enzymes and mitochondrial content [1, 2]. We measured the activity of two key oxidative enzymes that are indicative of muscle oxidative capacity, CS and SDH. Both are found in the mitochondria and are key enzymes of the Krebs cycle, and each has been demonstrated to increase in response to endurance training [3–7, 9, 10, 30].We also measured total protein content of the transcription coactivator peroxisome proliferator-activated receptor *γ* coactivator-1*α* (PGC-1*α*) as a marker for increased mitochondrial biogenesis. PGC-1*α* is a transcriptional coactivator of transcription factor PPAR*γ*, and together, they regulate the expression of genes that encode mitochondrial proteins. An acute bout of exercise or stimulated skeletal muscle contraction induces an increase in both PGC-1*α* mRNA and protein in skeletal muscle [31–34], and it has been shown that increased PGC-1*α* activation and total protein amount leads to increased mitochondrial biogenesis [24]. 

In the present study, we demonstrated that the activity of CS and SDH, and the total protein content of PGC-1*α* increased significantly in response to 4.5 wks of training. However, no significant treatment differences in these measures were detected. There was a slight but nonsignificant trend for a greater increase in CS and SDH activity in CM compared to CHO and PLA. It may be that the training period was not long enough to detect any potential differences that could emerge in response to chronic nutritional supplementation. Thus, our results suggest that the greater VO_2_ max improvements with CM supplementation are most likely due to cardiovascular adaptations rather than increases in oxidative enzymes or in mitochondrial biogenesis. 

As mentioned previously, endurance training leads to an adaptive increase in cardiac output, and this increase is due to augmented stroke volume [1]. While we did not measure these variables in the present study, our results suggest that the significant improvement in VO_2_ max in the CM group is likely due to increased stroke volume and cardiac output, which is likely due to increased plasma volume. Plasma volume expansion is a hallmark of aerobic endurance training [35] and is directly associated with increased plasma albumin content. Increased albumin in the plasma causes water to be retained in the vasculature due to increases in the colloid osmotic pressure gradient [36, 37]. Hepatic albumin synthesis has been shown to increase in response to endurance exercise training [38, 39]. Moreover, plasma albumin content was reported increased 23 h after an acute bout of cycling exercise when CHO+PRO supplementation was provided postexercise compared to placebo [40]. These results, along with the findings of the present study, suggest that hepatic albumin synthesis may have been increased to a greater extent in the CM group compared to the CHO or PLA groups and contributed to the significantly greater increase in VO_2_ max in the CM group.

Okazaki and colleagues [16] recently demonstrated that CHO+PRO supplementation provided immediately after daily cycling exercise training in older male subjects increased stroke volume and plasma volume compared to a placebo group. Their subjects cycled for 60 min/d, 3 d/wk for 8 wk at 60–75% VO_2_ peak and ingested either CHO+PRO or placebo immediately postexercise each session. VO_2_peak increased 3.3% in the control group and 6.8% in the CHO+PRO group, with significant stroke volume and plasma volume increases only found in the CHO+PRO group [16]. In the present study, we extend the findings of Okazaki and colleagues [16] by demonstrating that the effect of nutritional supplementation on VO_2_ max increases is nutrient specific. In comparing CM against an isocaloric CHO only supplement and a placebo, we have shown that the increased VO_2_ max response is not due to simply providing calories postexercise. In the present study, the VO_2_ max increase in the CHO and PLA groups was not significantly different. Thus, these results suggest that the benefit from a CHO+PRO or CM supplement in improving VO_2_ max is due to the combined ingestion of carbohydrate and protein. However, we cannot rule out the possibility that a supplement composed of protein alone would not have the same effect. 

In addition to well-documented increases in VO_2_ max with training, it is known that lactate threshold improves with endurance exercise training of moderate to high intensity [41]. In the current study, LT improved significantly over the 4.5 wks of training although there were no significant treatment differences detected (Table 4). It has been shown that the respiratory capacity of the muscle is the key determinant of LT [42]. Given that muscle oxidative enzyme activity and PGC-1*α* content increased significantly over time without demonstrating a treatment effect, it would be expected that LT would follow a parallel pattern. Therefore, the results suggest that while LT is increased by exercise training in parallel with muscle oxidative capacity, it likewise may not be affected by nutritional supplementation.

The other key finding of the present study was that body composition improvements, represented by a calculated lean and fat mass differential, were significantly greater in the CM group than the CHO group. Compared to the CHO group, the CM group lost more fat mass and gained more lean mass measured in the whole body, as well as in the trunk region only (*P* < .05). While these differentials were also greater for CM compared with PLA, the differences were not significant. 

It is well established that resistance exercise training induces significant gains in lean mass, whereas endurance exercise training is not associated with large increases in lean mass or gains in muscular strength [43]. A previous investigation comparing the effects of aerobic and aerobic + resistance training showed that the aerobic + resistance group increased lean mass in arm, trunk, and total body regions, and the aerobic only group increased lean mass in trunk region only [44]. However, the aforementioned investigation did not use supplementation. The body composition improvement with CM is also in agreement with the findings of Josse and colleagues [22], who recently demonstrated significantly greater muscle mass accretion, fat mass loss, and strength gains with milk supplementation compared to soy and CHO after a 12-wk resistance training program [22]. Therefore, the findings of our study are in line with what is reported in the literature for exercise mode-dependent body composition changes.

As shown in Table 5, all groups demonstrated significant changes over time in whole-body lean mass, trunk fat mass, and legs lean and fat mass, and the CM group demonstrated a significant treatment effect compared to CHO when whole body and regional differentials were calculated (Figures 4(a), 4(b), and 4(c)). The whole body and trunk differentials for PLA were slightly greater than CHO although not significantly different from either CM or CHO. The lack of difference in the PLA treatment from CHO suggests that a component of the CM treatment facilitated the significant body composition change, since simply supplementing with an energy-containing supplement (CHO) did not have a significant effect compared to PLA. In fact, a slight, nonsignificant increase in fat mass in the legs region was detected with CHO, whereas fat mass of the legs decreased in CM and PLA during the training period. To our knowledge, no evidence exists in the literature to suggest that postexercise CHO supplementation would mediate this type of change, given that the subjects' diets were not standardized and controlled during the study. However, this finding further underscores the difference in supplementing with a CHO+PRO-containing supplement versus calories from CHO alone in facilitating body composition changes.

There are two possible explanations for the difference found with the CM treatment compared to CHO: first, the availability of amino acids (AAs) in the milk for anabolism and muscle mass accretion, and second, a fat-loss promoting effect of dairy calcium and protein. It is known that AAs, along with a permissive amount of insulin, are required for muscle protein accretion to occur in response to exercise [45, 46]. The CHO treatment would increase plasma insulin levels and provide glucose as an energy and glycogen-synthesizing substrate, but would provide no AAs for the synthesis of new muscle protein. Thus, AAs availability from the milk proteins whey and casein provided substrate for this adaptive process. In addition, Zemel [47, 48] have shown that the increased consumption of dietary calcium is associated with reduced adiposity and greater weight loss in energy restricted diets. Moreover, the fat and weight loss effects were greater when the source of the dietary calcium was from dairy products rather than a calcium supplement [47, 48]. Additional evidence that the dairy component of the CM treatment likely underlies some of the body composition changes is found in the resistance training study of Hartman and colleagues [21], who demonstrated that fat mass decreased, and lean mass increased, in groups provided either milk, soy, or CHO postexercise but that milk significantly promoted increased hypertrophy compared to soy and CHO [21]. Another well-known benefit of dairy calcium consumption is improved bone mineral density. We did not detect treatment or time differences in BMD (Table 5); however, this is not surprising, given the relatively short duration of the training program and the lack of a resistance training component. Taken together, these data suggest that the dairy component of the CM treatment was instrumental in facilitating the fat mass changes compared to the CHO and PLA groups, while the AAs from milk proteins provided substrate for lean mass accretion in the present study.

There are several limitations to the present study. First, the subjects' normal diets were not controlled nor standardized for the majority of the training period. Although the diets were recorded and replicated for 3 days each week as described above, there could have been within and between-subject variations in the amount of protein, calcium, and total caloric intake on the nonrecorded days during the training period which could have influenced the adaptive response. Second, CM contains many other micronutrients and flavonoids in addition to the major macronutrients and calcium. However, the possible effects of these additional components on the training adaptations reported here are not known at this time. Third, the taste and appearance of the three treatments were different. However, the subjects were not aware of what the three treatments were, and since they only ingested the treatment for which they were randomized for the entire study period, they did not taste any of the other treatments. Fourth, we did not match the supplement dosing to each individual's body weight, but stratified the amount of supplement for each dose according to body weight ranges. We have previously shown that supplementation with ~1.0 g of CHO and ~0.3 g of PRO per kg body wt postexercise will substantially increase muscle glycogen synthesis and recovery from exercise [14]. In the present study, providing supplement based on a weight range represented a more realistic and practical approach. Finally, while we propose that the greater increase in VO_2_ max in the CM group is likely due to albumin synthesis, we did not measure plasma volume or plasma albumin and, therefore, cannot say with certainly that this is the reason for the VO_2_ max differences. Further investigation is necessary to expand upon these results and elucidate the mechanisms of the greater adaptive response.

## 5. Conclusion

Our results demonstrated that CM supplementation postexercise increased the magnitude of VO_2_ max improvement in response to a 4.5 wk aerobic exercise-training program. Muscle oxidative capacity and lactate threshold improved significantly in all treatment groups, with no differences found between treatments. This would suggest that the greater improvement in VO_2_ max when supplementing with CM as compared with CHO or placebo was cardiovascular rather than cellular in nature. In addition, CM supplementation significantly improved body composition as defined by the combination of an increase in lean mass and a decrease in fat mass compared to CHO. We conclude that CM is an effective postexercise recovery supplement that can induce positive increases in aerobic training adaptations in healthy, untrained humans.

## Figures and Tables

**Table 1:** Subject characteristics at baseline.

	All subjects (32)	CM (11)	CHO (11)	PLA (10)
Age (y)	22.0 ± 0.5	22.1 ± 0.7	21.3 ± 0.9	22.6 ± 1.0
Weight (kg)	71.7 ± 2.4	70.9 ± 5.1	71.2 ± 3.1	73.2 ± 4.5
Height (cm)	168.6 ± 1.5	169.1 ± 2.3	168.0 ± 2.7	168.8 ± 3.1
VO_2_ max (L·min^−1^)	2.6 ± 0.2	2.7 ± 0.3	2.6 ± 0.2	2.6 ± 0.2
VO_2_ max (mL·kg·min^−1^)	35.9 ± 1.9	36.8 ± 1.4	35.7 ± 2.2	35.2 ± 2.1

Values are mean ± SE. No significant differences existed between the groups at baseline.

Numbers in parentheses indicates subject numbers.

**Table 2:** Protocol for training period.

	Mon	Tue	Wed	Thurs	Fri	Sat	Sun
Baseline	LT and VO_2_ max testing; biopsy; DEXA scan						

Week 1 (75% VO_2_ max)	30 min	40 min	50 min	60 min	60 min	Rest	Rest
Week 2 (75% VO_2_ max)	60 min	60 min	60 min	60 min	60 min	Rest	Rest

Midpoint	VO_2_ max testing			60 min	60 min	Rest	Rest

Week 3 (75% VO_2_ max)	60 min	60 min	60 min	60 min	60 min	Rest	Rest
Week 4 (80% VO_2_ max)	60 min	60 min	60 min	60 min	60 min	Rest	Rest

End	LT and VO_2_ max testing; biopsy; DEXA scan						

**Table 3:** Energy and macronutrient content of the supplements.

	CM	CHO	PLA
CHO, g/100 mL	11.48	15.15	0
PRO, g/100 mL	3.67	0	0
Fat, g/100 mL	2.05	2.05	0
Kcals/100 mL	79.05	79.05	0
Ratio of CHO : PRO	3.12 : 1	—	—

Per 100 mL, CM, chocolate milk; CHO, carbohydrate + fat; PLA, placebo.

**Table 4:** Lactate threshold.

	Baseline	End
LT (VO_2_, L/min)		
CM	1.61 ± 0.16	1.83 ± 0.16^†^
CHO	1.47 ± 0.10	1.67 ± 0.11^†^
PLA	1.53 ± 0.11	1.70 ± 0.13^†^

Values are mean±SE. Significant differences: ^†^, time only (*P* < .05).

**Table 5:** Body composition.

	Baseline	End
Weight (kg)		
CM	71.7 ± 5.5	71.7 ± 5.5
CHO	71.4 ± 3.4	71.4 ± 3.4
PLA	73.2 ± 4.5	72.9 ± 4.4

Lean mass, whole body (kg)		
CM	49.6 ± 4.1	51.0 ± 4.1^†^
CHO	49.4 ± 3.7	50.0 ± 3.74^†^
PLA	47.7 ± 3.7	48.5 ± 3.5^†^

Fat mass, whole body (kg)		
CM	19.1 ± 2.2	17.7 ± 2.15^†^
CHO	19.0 ± 2.1	18.5 ± 1.9^†^
PLA	22.5 ± 2.6	21.5 ± 2.6^†^

Lean mass, trunk (kg)		
CM	23.7 ± 1.8	24.6 ± 1.7^§^
CHO	22.6 ± 1.7	22.7 ± 1.7
PLA	20.9 ± 1.6	21.3 ± 1.6

Fat mass, trunk (kg)		
CM	11.6 ± 1.6	10.7 ± 1.6^†^
CHO	10.2 ± 1.3	9.6 ± 1.1^†^
PLA	10.7 ± 1.3	10.0 ± 1.3^†^

Lean mass, legs (kg)		
CM	17.8 ± 1.4	18.3 ± 1.4^†^
CHO	16.7 ± 1.3	17.1 ± 1.3^†^
PLA	15.6 ± 1.2	16.0 ± 1.2^†^

Fat mass, legs (kg)		
CM	7.0 ± 0.9	6.6 ± 0.9^†^
CHO	6.7 ± 0.8	6.8 ± 0.8^†^
PLA	7.7 ± 0.8	7.5 ± 0.8^†^

Bone mineral density (g/cm^2^)		
CM	1.2 ± 0.1	1.2 ± 0.1
CHO	1.2 ± 0.0	1.2 ± 0.0
PLA	1.2 ± 0.0	1.2 ± 0.0

Values are mean ± SE. Significant differences: ^†^, significant time effect; ^§^, CM versus CHO (*P* < .05).
